# The Week's Work

**Published:** 1909-11-06

**Authors:** 


### The Week's Work.

### THE KING AT NORWICH HOSPITAL.

The Chairman of the Board of Management of the Norfolk and Norwich Hospital (the Rev. H. W. G. Thursby) has received the following letter from Mr. Haldane:—

[Copy.]

"War Office, 25th October, 1909.

"Dear Mr. Chairman,—The King commands me to express to you, and through you to those who organised the proceedings of to-day, his gratification with the arrangements made for the ceremony of laying the foundation stone of the extensions of the Hospital. His Majesty was much pleased with the progress the Hospital is making, and with the efficiency of its organisation.—Believe me, yours sincerely, (Signed) R. B. Haldane.

### THE BRADFORD SCANDAL.

For 15 years at least the Bradford Royal Infirmary has urgently required to be rebuilt. Like other northern cities Bradford has outgrown its infirmary, and the need for an up-to-date modern hospital has at length become recognised by the people. The Lord Mayor of Bradford with great public spirit has organised a special fund, and already raised £40,000 for the rebuilding of the Infirmary. With remarkable want of foresight, public spirit, and discretion, the authorities of the Bradford Eye and Ear Hospital have chosen the present moment, when more money is still required for the Infirmary, to issue an appeal for funds for their own institution. Similar indiscretions have been committed in the past in London, and have usually recoiled upon the offending institution. We hope that the people of Bradford will mark their sense of the ineptitude and impropriety of the action of the Eye and Ear Hospital by ignoring its appeal, at least until the whole sum required for the new infirmary has been subscribed. We would go a step further and say it would be to the public interest for the citizens to secure the amalgamation of the Eye and Ear Hospital with the Bradford Infirmary, so as to prevent any similar scandal in the future.

### "THE LOCAL GOVERNMENT REVIEW."

We have received a copy of this new monthly, which is published by Messrs. Simpkin, Marshall, Hamilton, Kent and Co. at the modest price of one shilling, and contains an abundance of interesting matter valuable to hospital workers, and those who are concerned with Poor Law and relief work generally. In this, the first number, we find Mrs.

ment," Mr. Dawson furnishing a singularly lucid and clear exposition of German methods in "German Municipal Government," while Dr. Kenwood pleads strongly for the unification of the public health service in another interesting and instructive article. These are only three of the many good papers in this issue, and we cannot pass over the excellent and practically helpful pages on Local Government Law and Practice, which are a feature of the paper. Such a monthly as this review promises to be is urgently wanted, and will be most useful to those who take an interest in Poor Law work and allied subjects, provided it maintains the high level of excellence which distinguishes its first issue.

### ENDOWED BEDS.

The Belgrave Hospital for Children has been the occasion of clearing up the following delicate question: "Supposing a hospital is left £1,000 to found a bed to be called after the testator, is it entitled to apply that legacy to its annual income, or is it under an obligation to treat the legacy as a capital sum, held in trust, the interest of which must be devoted to the maintenance of the bed in question?" Mr. Justice Swinfen Eady, in the High Court last week, decided that the Belgrave Hospital must not use as income the sum of £1,000 left for the "founding of a bed." Such legacies must be treated as capital sums for specific purposes, and if the sum is insufficient for the purpose, as was alleged in the case of the endowment of this bed, the legacy must be applied as far as it will go. No sane man will quarrel with this ruling. Intending benefactors will probably endorse his Lordship's expression of opinion that "it is of the greatest importance that when money is left for charitable purposes the terms of the gift shall be strictly adhered to." His Lordship further is reported to have said that "no suggestion was made of the fund not having been properly applied. It was only said that it (the legacy) had been used for income instead of capital." It is the custom to invest all sums given to a hospital for a definite purpose. Otherwise gifts of this kind would not be made. If a hospital undertakes to endow a bed for a definite sum the money must be devoted to that purpose only. This may make hospitals revise their scale of gifts for endowed beds.

### HOSPITALS AND LEGACY DUTIES.

There is no doubt that hospital treasurers, when they consider the demands made by our present standard of efficiency, are right in resenting any diminution of the gifts they receive by any form of State taxation. Their position is perfectly clear in the matter. So is the Government's position in keeping to the old principle that charitable bequests should not escape the legacy duties—however much we may disapprove of it. The only person whose standpoint is illogical and ridiculous is the benefactor. It is his business to know what is the sum he wishes any particular charity to receive, and equally how much he can afford to give to it. A moment's thought should remind him that what he pays in legacy duty—if he does pay it—is just as much a present to the charity as the gift itself. Whether he leaves money free of legacy duty or not, the result to the charity is exactly the same, except that if he does so leave it the charity is saved an incredible amount of time and vexation, points which ought to weigh more with a benefactor than the apparently, but only apparently, larger sum credited to his generosity in the newspapers, when the duty is not paid by himself.

### COMPENSATION CLAIMS AND HOSPITALS.

The action brought by a professional nurse against the Trustees and Committee of Management of the Royal Boscombe and West Hants Hospital (Mr. J. Cooper, J.P., and others) to recover under the Workmen's Compensation Act compensation in consequence of having contracted a septic thumb while dressing a hospital case, has drawn attention to the whole question of the liability of hospitals under the Act. Under the Acts of 1880, 1897, 1900, and 1906 it has generally been held that hospitals are liable in the case of workmen, and the term "workman" is usually held (Murray, *The Law of Hospitals*) to include nurses, though not necessarily sisters or matrons. The action was tried at the Bournemouth County Court before Judge Philbrick, K.C., and from the evidence led it appeared that the claimant has a permanent stiff thumb in consequence of the septic condition which was said to have been incurred through dressing. She is now incapacitated from following her occupation as a nurse. There had been no injury to the thumb such as a scratch or cut so far as she was aware, and when the condition had been diagnosed the thumb was operated upon at once.

### SOME POINTS IN THE CASE.

The argument for the defence submitted that legally there had been no proof of accident. The whole burden of proof that an accident occurred lay with the applicant as well as the fact that she had been disabled in consequence of such accident. Judge Philbrick stated that he was bound by a decision of the Court of Appeal in the Brodrick case, in which the applicant, who was employed in a sewer, and by his work necessarily exposed to the risk of sewer gas, contracted enteritis, and it was held that it was not an accident. He was exceedingly sorry to say that he was unable to distinguish between the two cases. He found there was no proof that the injury to the thumb was caused by accident, and that the case was covered by the decision in Brodrick's case. The application would therefore be dismissed, leave of appeal being granted. The decision appears to be in consonance with the best opinion on the subject, though in this particular instance there were several interesting points which will have to be more fully discussed on a future occasion. The case as it stands is a useful guide for future reference.

